# Millimeter-Wave Band Electro-Optical Imaging System Using Polarization CMOS Image Sensor and Amplified Optical Local Oscillator Source

**DOI:** 10.3390/s24134138

**Published:** 2024-06-26

**Authors:** Ryoma Okada, Maya Mizuno, Tomoaki Nagaoka, Hironari Takehara, Makito Haruta, Hiroyuki Tashiro, Jun Ohta, Kiyotaka Sasagawa

**Affiliations:** 1 Division of Materials Science, Graduate School of Science and Technology, Nara Institute of Science and Technology, 8916-5, Takayama, Ikoma 630-0192, Nara, Japan; okada.ryoma.on9@ms.naist.jp (R.O.);; 2 Radio Research Institute, National Institute of Information and Communications Technology, 4-2-1, Nukui-Kitamach, Koganei 184-8795, Tokyo, Japan; 3 Department of Opto-Electronic System Engineering, Chitose Institute of Science and Technology, 758-65, Bibi, Chitose 066-8655, Hokkaido, Japan; 4 Institute for Research Initiatives, Nara Institute of Science and Technology, 8916-5, Takayama, Ikoma 630-0192, Nara, Japan; ohta@ms.naist.jp; 5 Department of Health Sciences, Faculty of Medical Sciences, Kyushu University, 3-1-1, Maidashi, Higashi-ku 812-8582, Fukuoka, Japan

**Keywords:** electro-optic imaging system, millimeter-wave imaging, polarization image sensor, on-pixel polarizer, CMOS image sensor

## Abstract

In this study, we developed and demonstrated a millimeter-wave electric field imaging system using an electro-optic crystal and a highly sensitive polarization measurement technique using a polarization image sensor, which was fabricated using a 0.35-µm standard CMOS process. The polarization image sensor was equipped with differential amplifiers that amplified the difference between the 0° and 90° pixels. With the amplifier, the signal-to-noise ratio at low incident light levels was improved. Also, an optical modulator and a semiconductor optical amplifier were used to generate an optical local oscillator (LO) signal with a high modulation accuracy and sufficient optical intensity. By combining the amplified LO signal and a highly sensitive polarization imaging system, we successfully performed millimeter-wave electric field imaging with a spatial resolution of 30×60 µm at a rate of 1 FPS, corresponding to 2400 pixels/s.

## 1. Introduction

The visualization of the electric fields of millimeter waves is of considerable importance. This band is used for communication in 5th-generation mobile communication systems. In the research and development of wireless communication devices, the design, prototyping, performance testing, and assessment of the applicability to radio-frequency exposure guidelines are repeated to achieve the desired performance. Electric field imaging is expected to make a significant contribution to improving the efficiency of operational diagnostics and the electromagnetic field (EMF) compliance assessment of wireless communication devices.

In high-frequency electromagnetic field measurements, probes using antennas and coils have been widely employed, but metal elements and wiring are highly invasive and interfere with the measurement target [1,2,3,4]. Sensors with an array of antennas have also been studied [5,6]. This sensor can image the electric field image at once, but it is difficult to measure the near field for the same reason. Therefore, near-fields cannot be directly imaged, but must be inferred by simulation using data of the electric field measured from a distance [7]. In this method, the simulation time depends on the simulation model resolution. Furthermore, measurement using an antenna probe requires between 40 min to 2 h of scanning per measurement, making real-time evaluation impossible [8,9,10].

On the other hand, probes using electro-optic (EO) crystals based on high-frequency photonics enable minimally invasive and broadband measurements [11,12,13,14,15]. This method provides a highly sensitive measurement of the birefringence change of EO crystals due to electric fields. A method of obtaining electric field images by scanning a probe with an EO crystal attached to the end of an optical fiber has been proposed. This method enables electric field imaging with a high SNR because the amount of light that can enter the EO crystal is very high [16,17]. However, this method is very slow, due to the mechanical scanning required, and the imaging speed is about 3 pixel/s [18]. As a method that does not require mechanical scanning, a method to scan light with a Galvano mirror and capture the distribution of electric fields in EO crystals has been proposed. This method is capable of relatively high-speed imaging at 125 pixels/s [19]. However, faster imaging is required to observe signals that change in the time domain. The method combining EO crystals and image sensors does not require mechanical scanning or optical scanning, and enables the acquisition of electric field distribution in a single shot [20,21]. In this method, a single image is captured in a few seconds, which is very fast, but the optical limitations of the image sensor result in a low upper limit to the amount of light that can illuminate the EO crystals, which leads to poor sensitivity.

To detect weak polarization changes, it is necessary to limit the incident light intensity into the polarization image sensor to avoid pixel saturation, whereas it is necessary to illuminate large light intensity to the observation target. In our previous works, we proposed and demonstrated a method for measuring weak polarization changes using a polarization image sensor with a double-polarizer structure [20,21,22].

In this study, we set up a millimeter-wave-capable light source composed of an optical modulator and a semiconductor optical amplifier (SOA). Increasing the amount of light irradiating the EO crystal by optical amplification made it possible to improve the electric field measurement sensitivity based on the polarization change enchancement by the double-polarizer structure. Also, a differential amplification circuit was designed and included in a polarization image sensor to improve the signal detection performance. The differential amplification in the chip led to the wide sensitivity range of the sensor. We also demonstrated imaging of the millimeter-wave near-field, which was used in the 5th-generation mobile communication system, by combining these techniques. We successfully performed 30 GHz electric field imaging with a spatial resolution of 30×60 µm at a rate of 1 FPS, corresponding to 2400 pixels/s.

## 2. Electric Field Imaging System

### 2.1. Measurement Principle

**Figure 1:**
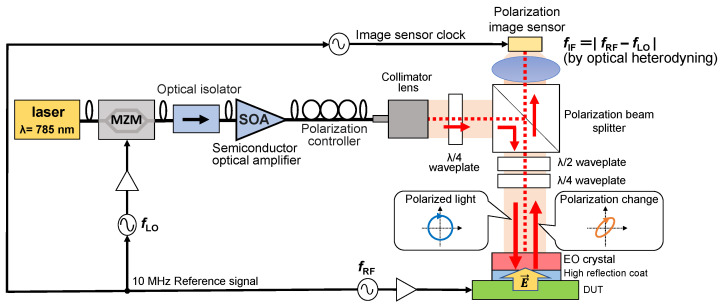
Conceptual diagram of the electric field imaging system.

Figure 1 shows a conceptual diagram of the electric field imaging system. In the EO measurement, electric field information is obtained by detecting the polarization change of light passing through an EO crystal, in which the birefringence is changed by the applied electric field owing to the first-order electro-optic effect.

In our previous works, to detect polarization changes with a high sensitivity, we proposed a method in which a polarization image sensor with on-pixel polarizers was combined with a signal-selective polarizer, as shown in Figure 1 [20,21,22]. Generally, the extinction ratio of on-pixel polarizers is lower than that of uniform polarizers [23,24,25]. The on-pixel polarizers of polarization image sensors with the 0.35-µm CMOS process cannot fabricate a polarizer pitch finer than the wavelength. When the polarizer pitch is wider than the wavelength, polarization is converted to light intensity based on light diffraction. This grating-based on-pixel polarizer has different polarization transmission characteristics compared with a normal wire-grid polarizer. Therefore, a wire grid polarizer shows a high transmittance when the grid is parallel to the polarization, but an on-pixel polarizer as a diffraction grating shows a high transmittance when the grid is vertical to the polarization. In general, the extinction ratio of this polarizer as a grating is lower than that of the wire-grid polarizer. To address this issue, the role of the polarizer is divided into “modulation enhancement” and “conversion of polarization modulation into light intensity modulation” in this setup. The former is achieved using a uniform polarizer. In this paper, we refer to this polarizer as a signal-selective polarizer. The latter is achieved using on-pixel polarizers. In this method, two types of on-pixel polarizers, 0° and 90°, are placed on the adjacent pixels to fabricate a polarization image sensor. The signal-selective polarizer is placed to be ±45° against these on-pixel polarizers. Here, the signal-selective polarizer significantly reduces the incident linear polarization. On the other hand, the orthogonal polarization component generated as a result of the birefringence of the EO crystal exhibits a high transmittance. As a result, the light intensity is considerably reduced and the degree of polarization change is enhanced.

The frequencies of micro and millimeter waves are much higher than the frame rate of the image sensor. Therefore, frequency conversion by the optical heterodyne method is applied to enable measurement with an image sensor. In this technique, the EO crystal works as an optical mixer. Thus, the frequency components of the electric field $f_{RF}$ and the intensity modulated light $f_{LO}$ are mixed and the intermediate frequency component $f_{IF}=\left|f_{RF}-f_{LO}\right|$ is generated. When the modulation amplitude of the incident light is constant, the amplitude of the intermediate frequency (IF) signal is proportional to the amplitude of the electric field intensity. By setting a sufficiently low $f_{IF}$ frequency, it is possible to measure the signal with an image sensor. The signal sources input to the MZM, RF signal source, and image sensor clock signal source are all synchronized by a 10 MHz reference signal. Therefore, the intermediate frequency and the image sensor frames were synchronized. As the image sensor was set at 360 FPS, the intermediate frequency was set at 90 Hz, which is 1/4 of the frame rate.

### 2.2. Setup of Electric Field Imaging System

**Figure 2:**
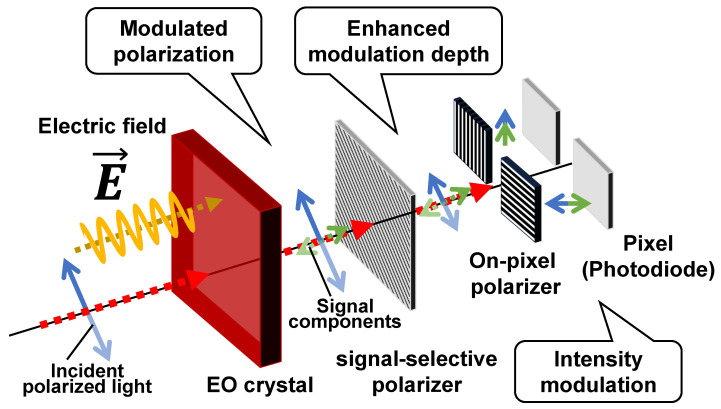
Polarization-imaging system based on the proposed method.

Figure 2 shows the setup of the electric field imaging system. For single-point EO measurements, the 1.55 µm band is often used. However, image sensors are usually made of Si and are sensitive to light from the visible to a near-infrared range of up to about 1 µm. Therefore, a single-wavelength CW laser (DBR785P, Thorlabs, Newton, NJ, USA) with a wavelength of 785 nm was used as the light source in this system. In this wavelength band, waveguide-based EO modulators and SOAs are available. In this study, a LiNbO_3_-based Mach–Zehnder modulator (MZM) (NIR-MX800-LN-20, iXblue Photonics, Paris, France) is used for optical intensity modulation.

Another important device is the SOA. The intensity launched into an MZM in this wavelength range is limited up to several mW owing to the photorefractive effect. In this work, the light intensity was increased by introducing an SOA. As shown in Figure 2, after passing through an optical isolator, the modulated light is amplified by a high-gain SOA (SOA-780-20-YY-30dB, Innolume, Dortmund, Germany) and enters the optical imaging system. The combination of MZM and SOA enables the generation of high frequency and high intensity LO optical signals. The maximum output power from the SOA was 41.5 mW. The light was collimated by a collimator to a beam diameter of 7.5 mm and launched to the optical system. When imaging was performed under these conditions, the output of the image sensor was about 15% of the pixel saturation level. As there is still some margin in the light-receiving level of the image sensor, SNR would be improved by using a higher-gain SOA.

In this optical system, the polarization beam splitter works as a signal-selective polarizer. The quarter-wave plates and half-wave plates were used to adjust the polarization state. The EO crystal used as the electric field probe was (100)-ZnTe, which responds to the electric field perpendicular to the plane [26]. In this setup, the EO crystal was placed directly on top of and in contact with the device under test (DUT) for near-field imaging. To reduce the invasiveness to the near-field or avoid physical interference, a floating arrangement of the EO crystal is also possible. However, the spatial resolution and field signal strength are reduced. The proposed method enables the visualization of millimeter-wave electric fields with a high sensitivity by combining a highly sensitive polarization imaging system and an electric field imaging method based on the EO effect.

## 3. Polarization Image Sensor with Differential Amplifiers

### 3.1. Specifications

**Figure 3:**
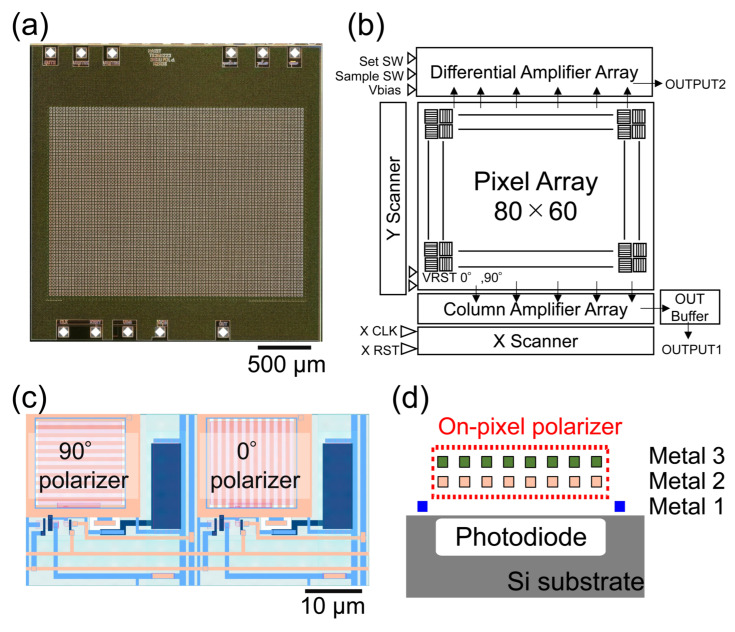
(**a**) Chip photograph. (**b**) Block diagram. (**c**) Pixel layout. (**d**) Cross-sectional view of pixel layout.

An image sensor equipped with on-chip polarizers was fabricated using a 0.35-µm 2-poly 4-metal standard CMOS process. Table 1 shows the sensor specifications. Figure 3 shows (a) a photograph of the fabricated polarization image sensor chip, (b) a block diagram of the image sensor, (c) the pixel layout, and (d) the cross-sectional view of the pixel structure. To achieve the configuration shown in Figure 1, each adjacent pixel is equipped with two types of polarizers that are mutually orthogonal to each other. The on-pixel polarizers are designed with the second (M2) and third (M3) metal wiring layers of the CMOS process to achieve a high extinction ratio. Here, the polarizer patterns of these two layers are identical. It is different from those in our previous work [22]. For high-sensitivity electric field imaging, the image sensor with the best performance in this wavelength band should be used. As described in the previous section, we chose the 780 nm wavelength band for electric field imaging, which is detectable by Si-based image sensors and can be used for high-speed optical modulators and amplifiers. By taking the difference between $0^{{}^{\circ}}$ and $90^{{}^{\circ}}$ pixel pairs, the differential detection can be achieved by the sensor itself, which reduces the common mode noise and improves the signal-to-noise ratio (SNR). In our previous works, data processing to obtain the differential signals was performed on a computer. On the other hand, differential amplifiers were integrated into this chip.

### 3.2. Pixel Characteristics

#### 3.2.1. Signal-to-Noise Ratio

**Figure 4:**
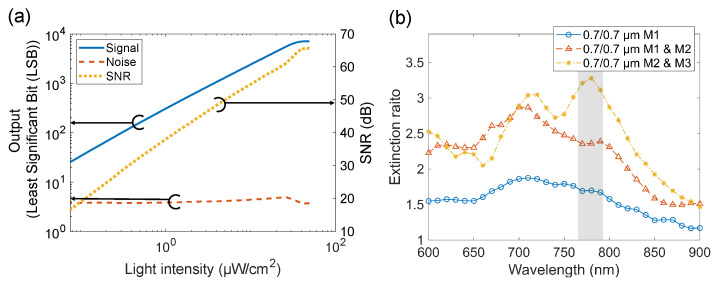
(**a**) Signal, noise, and SNR of the pixel. (**b**) Extinction ratio spectra of the dual-layer grating structure.

Because the signal intensities obtained by EO measurement were very low, an image sensor with a high SNR was required to detect weak polarization changes. The SNR measurement results are shown in Figure 4a. We performed imaging using a 14-bit ADC in the 0 to 2 V range. The measured maximum SNR was approximately 61 dB. This result indicates that an optical signal change of less than 0.1% could be detected when the incident light level was sufficient.

#### 3.2.2. Extinction Ratio of On-Pixel Polarizers

We evaluated the extinction ratio characteristics of the dual-layer structure of on-pixel polarizers. We compared three types of metal grid polarizers: a polarizer using only the first layer (M1), a polarizer using M1 & M2, and a polarizer using M2 & M3, which was not investigated in our previous work [22]. A polarizer (LPVIS100-MP2, Thorlabs) was mounted on the spectrometer and the extinction ratio at each wavelength was measured. The results are shown in Figure 4b. The extinction ratio of the structure using M2 & M3 exceeded 2.5 over a wide wavelength range from 690 to 810 nm, with a peak extinction ratio at 780 nm of 3.27. Extinction ratio spectra depend on metal layer combinations, probably because of the difference in the resonance condition between the metal layers. Compared with our previously fabricated image sensors, the extinction ratio was improved by increasing the number of polarizer layers from one to two, as well as by changing the arrangement of the two polarizer layers [20,21,22].

### 3.3. Differential Amplifier Performance

**Figure 5:**
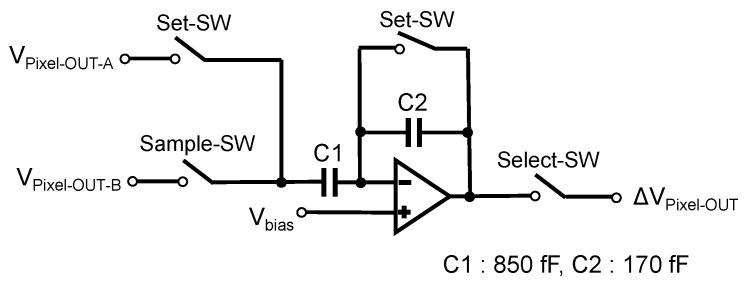
Circuit diagram of the differential amplifier.

In addition to the normal pixel output, this chip integrates a differential amplifier that amplifies and outputs the difference between the 0°- and 90°-pixel outputs. The circuit diagram of the differential amplifier mounted on the sensor is shown in Figure 5. The bias component due to the difference in characteristics between the 0° and 90° pixels was corrected by adjusting the pixel reset voltages for the 0° and 90° pixels. One differential amplifier was placed for each pair of two rows, and the entire chip consisted of an array of 40 differential amplifiers. The differential amplifier was composed of a switched capacitor circuit with capacitors to provide ×5 gain. In our previous system, the subtraction between columns was done in software after image acquisition. However, by performing this process in-chip, noise can be reduced.

**Figure 6:**
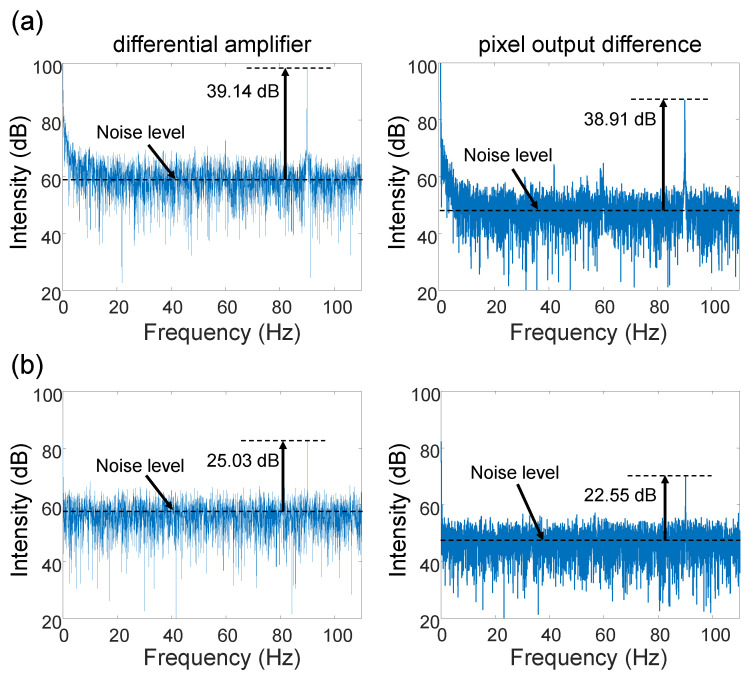
EO signal spectrum obtained from the differential amplifier and pixel output difference of (**a**) high incident light intensity (29.4 mW) and (**b**) low incident light intensity (1.2 mW).

To examine the performance of the differential amplifier, we carried out electric field imaging and compared the results with those obtained by the conventional software subtraction method. The system shown in Figure 1 was used. The DUT was a microstrip line. The frequencies were set to $f_{RF}$ = 100 MHz + 90 Hz and $f_{LO}$ = 100 MHz. Thus, the IF was 90 Hz. Measurements were taken at 360 frames/s, and 10,000 frames were acquired. The results were pixel averaged over 2×2 pairs of polarizers on the line with the highest electric field intensity. Here, the electric field intensity was obtained by fast Fourier transform (FFT). The modulated light intensity was set to 29.4 mW (high) or 1.2 mW (low). The results are shown in Figure 6a,b.

When the incident light level was 29.4 mW, both the differential amplifier and the pixel output difference methods showed similar SNRs. Next, the incident light was set to 1.2 mW, which corresponded to a 14 dB reduction. As shown in Figure 6b, the SNR of the differential amplifier setup was reduced by 14.1 dB, which was almost the same amount as the light intensity reduction. On the other hand, the SNR calculated from the pixel output difference was reduced by 16.4 dB. Signal amplification by the differential amplifier reduced the effects of noise caused by the subsequent readout circuitry, and relaxed the requirement of the light intensity needed for detecting EO signals. The electric field imaging system assumed that the measurement was made in a region where the incident light intensity was sufficiently high, i.e., where the photon shot noise was higher than the readout noise. However, in actual optical systems, even if sufficient light intensity is obtained near the center, it may be insufficient at the periphery of the imaging range. Expanding the range of acceptable light intensity reduces the optical design requirements for field imaging systems and allows for the use of smaller optical systems. In addition, as differential detection is performed in the image sensor, the speed of the ADC can be reduced by half, thus relaxing the requirement for ADC performance. With a small EO crystal as in this case, the non-uniform distribution of irradiated light intensity is not a significant problem. However, this problem is expected to be more pronounced when large EO crystals are used for large-area field imaging. At low light levels, the noise associated with signal readout became dominant, resulting in a low SNR. Differential amplifiers improved SNR by amplifying the signal within the image sensor chip. Furthermore, by acquiring the difference between 0° and 90° pixels, the common-mode noise component due to variations in incident light intensity could be greatly reduced, enabling imaging with a high SNR [20].

## 4. Demonstration of 28 GHz Microstrip Line Electric Field Imaging

### 4.1. Optical LO Signal Source

Two methods of generating local oscillation modulation [27] in the 28 GHz millimeter-wave band were investigated: one is to supply a 28 GHz signal to the optical modulator and perform double-sideband (DSB) modulation to produce 28 GHz intensity-modulated light, and the other is to supply 14 GHz signal to the optical modulator and perform double-sideband suppressed-carrier (DSB-SC) modulation by adjusting the bias point and producing 28 GHz modulation with the ±1st-order sidebands.

**Figure 7:**
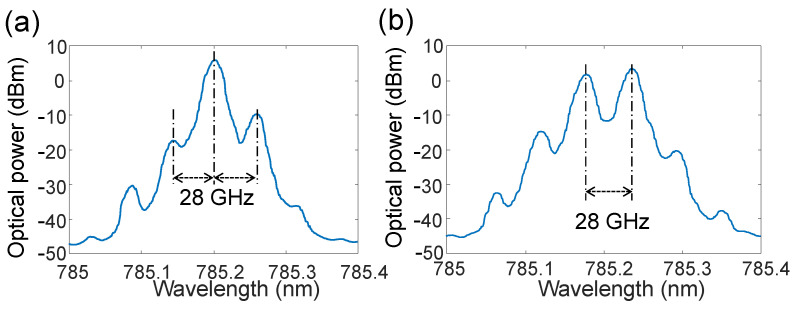
Optical spectra obtained from optical LO signal source of (**a**) 28-GHz DSB modulation and (**b**) 14-GHz DSB-SC modulation.

Figure 7a,b show the results of DSB modulation at 28 GHz and DSB-SC modulation at 14 GHz, respectively. The CW laser output was modulated by MZM, passed through the optical isolator, and amplified by SOA. Then, the modulation condition was measured with an optical spectrum analyzer. Both DSB and DSB-SC modulation were measured with the same SOA gain. The wavelength resolution was set to 0.01 nm, which corresponded to approximately 5 GHz. The 28 GHz DSB modulation results showed sidebands from the carrier wave to the left and right at a position corresponding to 28 GHz. For the 14 GHz DSB-SC modulation, the carrier component was well suppressed compared with the sideband component, and the sidebands could be observed at a position corresponding to 28 GHz between the sidebands. Theoretically, the two modulation schemes provided similar signal strengths under optimal modulation conditions. However, in this study, the 3 dB modulation bandwidth of the optical amplifier was 20 GHz, making it difficult to achieve higher modulation with DSB. In addition, the double-polarizer optical system used in this study was characterized by its ability to avoid pixel saturation of the image sensor and to achieve a high sensitivity using high-intensity optical LO signals.

Different from the previous study [27], we introduce SOA in this study to take advantage of the above feature. In DSB-SC modulation, even if the modulation signal intensity was somewhat lower than the optimum point, the ±1st order sidebands became the largest wavelength component, and high modulation could be obtained. Therefore, efficient optical amplification was possible and suitable for this system.

### 4.2. Imaging Target and Imaging Setup

The DUT was a microstrip line formed on a high-dielectric-constant substrate with a dielectric constant of approximately 10.2. The EO crystal in this experiment was 3×3×0.3 mm (100)-ZnTe. The RF signal input to the DUT was a 28 GHz + 1 kHz signal with AM modulation at 910 Hz because the setting resolution of the signal generator was 1 kHz. With this setting, the frequency component of 28 GHz + 90 Hz was generated. The optical LO signal was set at 28 GHz. The IF signal at 90 Hz was observed using the image sensor at a frame rate of 360 frames/s.

Data from the differential amplifier output for 3600 frames were processed to obtain the electric field image. It took 10 s to capture one image of the electric field—thus, 240 pixel/s. The IF component at 90 Hz on each pixel was extracted by FFT. By combining the FFT results, the intensity and phase distribution images were reconstructed.

### 4.3. Imaging Performance Comparison between DSB-SC Modulation and DSB Modulation

**Figure 8:**
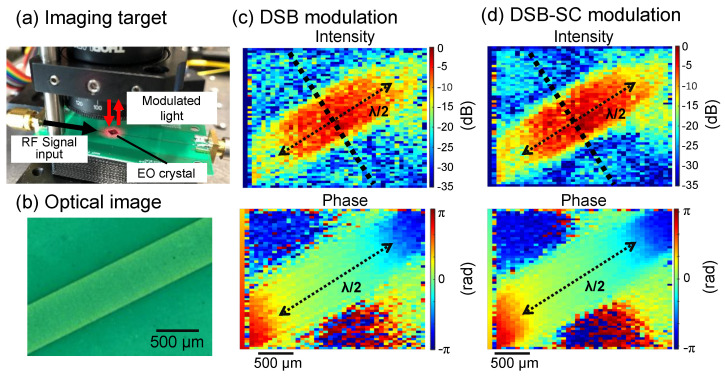
(**a**) Photograph of the imaging target. (**b**) Optical image of DUT. EO imaging results with (**c**) 28-GHz DSB modulation and (**d**) 14-GHz DSB-SC modulation.

Electric field imaging was carried out with the setup shown in Figure 8a. An EO crystal with a high-reflection coating on the bottom and an anti-reflection coating on the top was placed on the DUT. Figure 8b shows the photograph of the microstrip line as the DUT; the line width of the sample was approximately 0.56 mm.

The obtained intensity images with 28 GHz DSB modulation and 14 GHz DSB-SC modulation are shown in Figure 8c,d, respectively. The imaging results of the two modulation methods are almost the same. The electric field imaging results were normalized to display the maximum value as 0 dB. Comparisons using a calibrated EO probe were considered to be possible for converting the results to electric field strength and power density values. The EO crystal used in this experiment ((100)-ZnTe) was sensitive to the electric field vertical to the DUT [26]. The electric field spread upward from the microstrip line, swung around, and returned to the ground on the back of the PCB. As a result, we confirmed that the electric field intensity was higher directly above the line, lowest at the edge of the line, and lower outside the line, and the phase difference between the inside and outside of the line was π rad. This was in agreement with previous reports [28,29]. The DUT in this study caused signal reflections at the connector ends, and standing waves were observed in the line. Wavelength shortening occurred inside a substrate with a high dielectric constant of 10.4. The wavelength-shortening ratio was about 0.36, and the signal wavelength at 28 GHz was estimated to be about 3.85 mm. Half of the wavelength was about 1.93 mm. This result almost matched the wavelength of the standing wave in the image.

**Figure 9:**
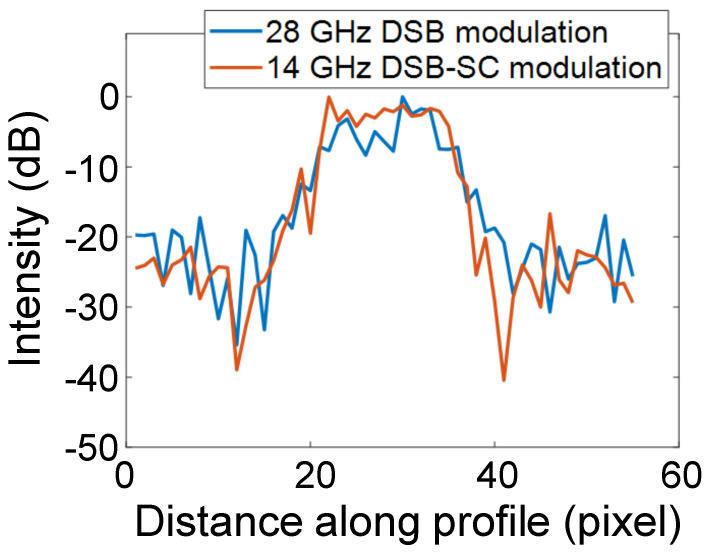
Profiles along the black dotted lines for 28-GHz DSB modulation in Figure 8c and 14-GHz DSB-SC modulation in Figure 8d.

The intensity profiles along the black dotted lines in Figure 8c,d are shown in Figure 9. The results show that a higher SNR was achieved with DSB-SC modulation. This may be due to the lower level of DSB modulation, as seen from the optical spectrum in Figure 7. When observing higher frequencies, the optical modulator and amplifier need to be compatible with higher frequencies. On the other hand, in this study, an optical intensity modulator was designed for 20 GHz, and DSB-SC modulation at 14 GHz was used to observe 28 GHz. The results suggest that optical LO signal generation using the harmonics of the optical modulator can be used for even higher frequencies.

## 5. Demonstration of 30-GHz Patch Antenna Sequential Electric Field Imaging

### 5.1. Imaging Target, Setup, and Evaluation of Electric Field Imaging Speed

**Figure 10:**
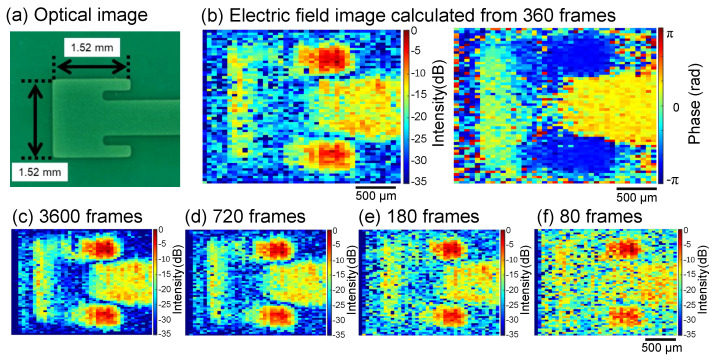
(**a**) Optical image of 30 GHz patch antenna. (**b**) EO imaging results of intensity distribution and phase distribution calculated from 360 frames. EO imaging results calculated from (**c**) 3600, (**d**) 720, (**e**) 180, and (**f**) 80 frames.

The DUT was a patch antenna fabricated on a high-dielectric-constant substrate with a dielectric constant of approximately 10.2, as shown in Figure 10a. This patch antenna is designed for 30 GHz. The EO crystal in this experiment was 3×3×0.1 mm (100)-ZnTe. The use of thin crystals reduces the optical path length, which decreases sensitivity, but it improves spatial resolution. The RF signal was set to 30 GHz + 90 Hz using a frequency doubler, amplified to approximately 28 dBm by an RF amplifier, and input to the DUT. Different from the previous experiment, the signal source used in this experiment has an upper frequency limit of 20 GHz, but can be tuned at 0.01 Hz. By inputting a 15 GHz signal to the MZM, 30 GHz DSB-SC modulation was performed to generate an optical LO signal. The IF signal at 90 Hz was observed using the differential amplifier output of the image sensor at a frame rate of 360 frames/s.

Figure 10b–f show the results of performing the FFT by changing used frames and reconstructing the electric field image. Figure 10b shows the intensity distribution and phase distribution of the electric field image reconstructed from 360 frames. Figure 10c–f show the electric field intensity distribution calculated from 3600, 720, 180, and 80 frames. It can be seen that the noise level decreased as the number of frames increased, resulting in a clear image of the electric field. Because of the frame rate of the image sensor and the upper limit of the amount of light that the SOA can output, the result of the calculation from 360 frames in this setup was considered to be a good balance between image quality and imaging speed. Each electric field image took 1 s, which enabled electric field imaging at a rate of 2400 pixels/s. This imaging speed was much faster than electric field imaging systems that use mechanical or optical scanning. Compared with the previously reported optical scanning system, the acquisition speed of our system was 19 times faster [19]. The spatial resolution for mechanical scanning was determined by the probe size, and for optical scanning, it was determined by the optical scanning resolution, represented by the beam diameter and the angular resolution of the galvano mirror. In our imaging setup, the spatial resolution was approximately 30×60 µm.

In this setup, the irradiated light intensity was about 15% of the pixel saturation level, leaving room for improving sensitivity by increasing the light intensity. Therefore, using a more higher-gain optical amplifier such as a tapered SOA improved SNR and enabled even faster imaging. In addition, an increase in the image sensor frame rate enabled an increase in the number of images accumulated per unit time, which improved the SNR and was expected to increase the electric field imaging frame rate. Furthermore, the higher frame rate shortened the exposure time, allowing the image sensor to detect a larger amount of incident light, which, when, adjusted to the same signal level of incident light, significantly improved the SNR, enabling faster and more sensitive imaging. The intermediate frequency could also be improved as the imaging frame rate increased. Due to the imaging system, the intermediate frequency was set at a very low frequency of 90 Hz, but this frequency band was highly susceptible to mechanical noise. More stable field imaging is possible by increasing the intermediate frequency from a several KHz to several MHz [30]. This high-speed imaging is difficult to achieve simply by improving the image sensor clock, so it is possible to achieve this by using an image sensor that stores pixel output [31,32,33,34]. We studied this in order to improve the intermediate frequency by controlling the exposure time while keeping the frame rate of the image sensor the same, but this technique took a long time to capture a single electric field image [35]. In addition, the resonant structure of EO crystals can dramatically improve sensitivity, although the frequency bandwidth that can be measured is narrower [29,36].

### 5.2. Sequential Electric Field Imaging Results

**Figure 11:**
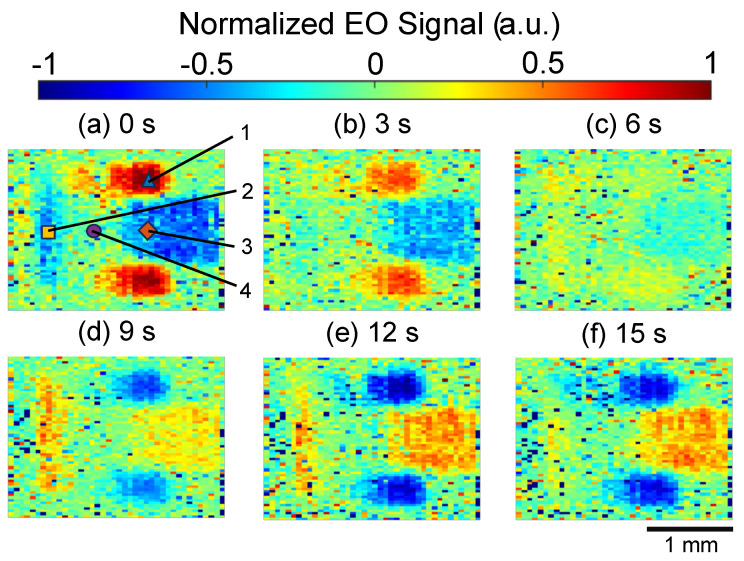
Sequential electric field distribution image on the patch antenna from 0 to 15 s.

In 30 GHz patch antenna electric field imaging, sequential electric field images were acquired by performing long duration imaging and computing each 360 frame sequentially. By setting the intermediate frequency slightly off the frequency of the numerical signal processing used to reconstruct the electric field image, it can be swept continuously [37]. The signal input to the patch antenna was set to 30 GHz + 90.04 Hz. This makes it possible to observe the electric field image through phase rotation by 0.04 Hz by reconstructing the electric field image at 90 Hz. In order to combine the phase and intensity information into a single image, the normalized EO signal was calculated from each field image as Acosθ, where *A* is the normalized intensity of the field intensity and θ is the measured phase. Figure 11 shows the normalized EO signal distribution at 3 s intervals from 0 to 15 s. As time progressed, the phase of the input signal rotated, so the normalized EO signal decreased and reappeared after sign inversion.

**Figure 12:**
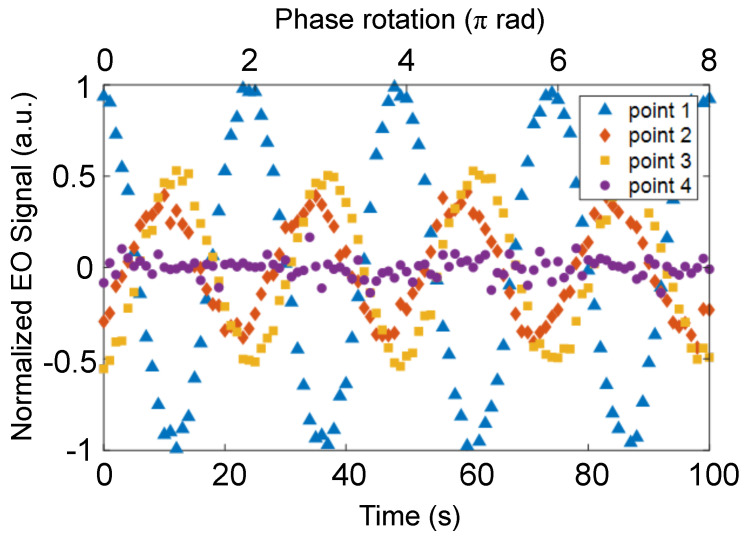
Normalized electrooptic signal intensity at points 1–4 indicated in Figure 11.

Figure 12 shows a time plot of the normalized EO signal intensity for points 1–4 shown in Figure 11. In this figure, the results are shown from 0 s to 100 s. As the electric field image was generated at 1 FPS and the phase was rotated at 0.04 Hz, a phase change of 8π in 100 s could be observed. The normalized EO signals were along sinusoidal curves because the input signal phase varied linearly with time. The (100)ZnTe was sensitive to the electric field in the vertical direction from DUT. Therefore, it could be observed that the electric field strength at the corner of the patch antenna and on the microstrip line was stronger, and the phase was shifted by π. The EO signal strength at point 4 was very weak because the electric field strength at the center of the patch antenna was weak.

## 6. Conclusions

In this study, we developed a system for the near-field imaging of millimeter waves. Our proposed high-sensitivity polarization imaging technique was introduced into the EO imaging system, and imaging of the electric near-field at 28 GHz and 30 GHz was performed.

By introducing a differential amplifier circuit into the polarization image sensor, we confirmed that analog subtraction and amplification could be performed inside the chip and that the performance degradation rate could be suppressed at low light intensities. In addition, to obtain a high performance in our recently proposed dual-polarization optical system, we verified the method of amplifying the modulated light generated by a CW laser and an optical modulator using SOA and confirmed that a higher performance could be obtained with DSB-SC modulation. In the electric field imaging of the 30 GHz patch antenna, we successfully imaged the electric field image at 1 FPS and sequentially imaged the phase change at 0.04 Hz. Electric field imaging is possible at a rate of 2400 pixels/s, which is much faster than electric field imaging systems that use mechanical or optical scans.

This electric field imaging system allows EO crystals to be placed near the antenna of a smartphone or other device to image the nearby electric field in a short period of time. Therefore, the system enables multifaceted evaluation by performing electric field imaging under conditions close to actual use, such as when the antenna is mounted on housing. Although the sensitivity is slightly lower than that of single-point electric field imaging, the spatial resolution and imaging speed are extremely fast, enabling imaging that follows time-varying signals. In particular, by feeding back the electric field frequency of the device-under-test to the LO system, it is expected to be possible to image the electric field in various communication conditions, such as frequency modulation [38].

It is known that the EO crystal responds to the THz band [12]. By using broadband modulation techniques and synchronizing the IF with the frame of the image sensor, it will be possible to extend the observation of these very high-frequency electric fields [39,40,41].

## Figures and Tables

**Table 1:** Image sensor specifications.

Technology	0.35-µm 2-poly 4-metal standard CMOS
Pixel size	30 × 30 µm
Photodiode size	15 × 15 µm
Photodiode	n-well/p-sub
Pixel type	3-Tr active pixel sensor
Number of pixels	80 × 60 (40 × 60 pairs)
On-pixel polarizer structure	Line/Space = 0.70/0.70 µm (M2 & M3)
Extinction ratio	3.27 (0°), 3.31 (90°) at 780 nm
Responsivity	0.98 @780 nm (Peak wavelength: 750 nm)
NEP	$1.5\times 10^{-13}$ (W/$\sqrt{\mathrm{Hz}}$ /pixel) @780 nm

## Data Availability

Data underlying the results presented in this paper are not publicly available at this time but maybe obtained from the authors upon reasonable requests.
